# Induction of CD4^+^CD25^+^ Regulatory T Cells from In Vitro Grown Human Mononuclear Cells by Sparteine Sulfate and Harpagoside

**DOI:** 10.3390/biology9080211

**Published:** 2020-08-06

**Authors:** Nour Z. Atwany, Seyedeh-Khadijeh Hashemi, Manju Nidagodu Jayakumar, Mitzi Nagarkatti, Prakash Nagarkatti, Mona Rushdi Hassuneh

**Affiliations:** 1 Department of Applied Biology, Faculty of Science, University of Sharjah, Sharjah 61467, UAE; u16101505@sharjah.ac.ae (N.Z.A.); u00038492@sharjah.ac.ae (S.-K.H.); 2 Sharjah Institute for Medical Research (SIMR), University of Sharjah, Sharjah 61467, UAE; mjayakumar@sharjah.ac.ae; 3 Department of Pathology, Microbiology, and Immunology, School of Medicine, University of South Carolina, Columbia, SC 29208, USA; mitzi.nagarkatti@uscmed.sc.edu (M.N.); prakash@mailbox.sc.edu (P.N.)

**Keywords:** human, Foxp3 induction, CD4^+^CD25^+^ regulatory T cells, natural molecules, mixed lymphocyte reaction (MLR)

## Abstract

Regulatory T cells (Tregs) are key players in the regulation of inflammatory responses. In this study, two natural molecules, namely, sparteine sulfate (SS) and harpagoside (Harp), were investigated for their ability to induce Tregs in human peripheral blood mononuclear cells (PBMCs). PBMCs were isolated from healthy volunteers and grown in the presence or absence of ConA, with TGF-beta, SS or Harp. Expression of the mRNA of FoxP3, TGF-beta, IL-10 and GAPDH was assessed via q-PCR. The expression of Treg markers including CD4, CD25, CD127 and FoxP3 was measured via flow cytometry. The secretion of IL-10 and TGF-beta by cultured cells was assessed by ELISA. Furthermore, the suppressive role of SS and Harp on PBMCs in vitro was tested via allogeneic mixed lymphocyte reaction (MLR). Data obtained show that both compounds increased the expression of FoxP3, TGF-beta and IL-10 mRNA in resting PBMCs but to a lesser extent in activated cells. Moreover, they significantly increased the percent of CD4^+^CD25^+^FoxP3^+^CD127^−^ Tregs in activated and naïve PBMCs. Functionally, both compounds caused a significant reduction in the stimulation index in allogeneic MLR. Together, our data demonstrate for the first time that SS and Harp can induce human Tregs in vitro and therefore have great potential as anti-inflammatory agents.

## 1. Introduction

Regulatory T cells (Tregs) are considered a specialized subset of CD4^+^ T cells that plays a major role in the establishment and maintenance of immune tolerance. Since their discovery in 1969 [1], Tregs have been extensively studied and identified as a promising potential therapeutic tool, especially to prevent exaggerated immune responses, autoimmunity and graft rejection [2,3,4,5,6,7]. Since then, several types of Tregs have been described; the main 3 types of CD4^+^ Tregs are: IL-10-producing type 1 regulatory T (Tr1) cells, TGF-β-producing CD4^+^ T helper 3 (Th3) cells and CD4^+^CD25^+^Foxp3^+^ T cells. Both Tr1 and Th3 lack fork-head box P3 (Foxp3) expression, and several cytokines were shown to account for their inhibition. The transcriptional factor Foxp3 is essential for the development of Tregs in the thymus, and loss of FoxP3 expression can result in autoimmune pathology that may affect multiple tissues and organs [2,8,9]. The expression of Foxp3 has been found to be related to the development and function of most Tregs, including the subpopulation that develops in the thymus (nTregs) and the subpopulation peripherally induced in secondary lymphoid organs (iTregs) [10,11]. Helios is a member of the Ikaros transcription factor family. It has been proposed that expression of Helios distinguishes nTregs from iTregs [12]. Treg-mediated suppressive activity involves various molecules including cytotoxic T-lymphocyte-associated protein 4 (CTLA-4), IL-2, IL-10, transforming growth factor-β (TGF-β), IL-35, glucocorticoid-induced TNF receptor (GITR), lymphocyte-activation gene 3 (LAG3), granzyme B, adenosine and cyclic AMP (cAMP) [3,13,14,15,16,17]. TGF-β plays a key role in maintaining the regulatory function of Tregs, as was shown in mice when anti-TGF-β was administered, which resulted in development of severe colitis [18]. iTregs are induced from naïve CD4^+^ T cells in the periphery and require the FoxP3 pathway; they are characterized as being CD4^+^CD25^+^FoxP3^+^CD127^−^ [19,20]. Tregs were first induced in vitro using TGF-β in 2003 by Chen et al. [11]. Tregs can be used to restore balance to immune cell functions and suppress inflammation in patients with autoimmune or chronic inflammatory disorders [17]. Several natural compounds have been shown to induce FoxP3 expression and promote a Treg cell phenotype [21,22,23], with the potential to suppress chronic inflammation associated with multiple clinical disorders.

In the current study, two compounds that were identified in a small molecule library screening for FoxP3 promoter induction were investigated for their ability to induce human Tregs from naïve and activated peripheral blood mononuclear cells (PBMCs) in vitro. The two compounds were sparteine sulfate (SS), which is an alkaloid found in *Lupinus* species and other Leguminosae, and harpagoside (Harp), an iridoid glycoside found in extracts of the plant *Harpagophytum procumbens*, also known as devil’s claw. SS has been shown to be useful as an antiarrhythmic, oxytocic and anticonvulsant [15,22,24,25,26,27], while Harp has been shown to have analgesic and anti-inflammatory properties [28]. Despite the reported medicinal uses, this is the first study to assess their impact on T cells and their ability to induce Tregs in vitro. The two compounds are nontoxic and effective in small doses, as demonstrated in this study, and could prove significant in the search for treatment for autoimmune and inflammatory pathologies.

## 2. Materials and Methods

### 2.1. Donor Blood

Healthy volunteer blood specimens were drawn in accordance with University of Sharjah Research Ethics Committee (REC) regulations. All subjects provided written informed consent prior to their blood donation. The donors were healthy middle-aged adults (3 females and 1 male, aged 25–50 years). The selection criteria were as follows: not ill at the time of the sample collection; no history of chronic or autoimmune disease; not taking any medications for the last 5 days; all were HIV and HBV negative; females were not pregnant. Blood was drawn by trained and certified personnel, and from each volunteer, about 50–60 mL was collected in a BD vacutainer tube containing lithium heparin as an anticoagulant.

### 2.2. Reagents

Lithium heparin vacutainers were purchased from BD Biosciences (San Jose, CA, USA); Tissue culture medium RPMI, fetal bovine serum (FBS), penicillin-streptomycin and phosphate buffered saline (PBS) from Gibco, Thermo Fisher Scientific (Dublin, Ireland); Concanavalin A (Con A) (Cat# sc-203007A), XTT tetrazolium sodium salt (CAS 111072-31-2) (Cat# sc-258336), harpagoside (CAS number: 19210-12-9) (Cat# sc-203073) and sparteine sulfate (CAS number: 299-39-8) (Cat# sc-471860) from Santa Cruz Biotechnology (Santa Cruz, CA, USA); human recombinant TGF-β (Cat# 580702) from BioLegend (San Diego, CA, USA); Mitomycin C (Cat# M4287) and Histopaque^®^-1077(Cat# 10771) from Sigma-Aldrich (St. Louis, MO, USA).

### 2.3. Natural Molecule Discovery

The Natural Products Collection library (NP120801) was purchased from MicroSource Discovery Systems, with an 800-molecule collection in 96-well format. Each plate was diluted to 1 μM and 0.1 μM in complete DMEM medium with 10% FBS and containing 3 μg/mL puromycin. The HEK 293 Human FoxP3 Prom/LUCPorter™ stable reporter cell line (IMGENEX) was plated at 0.1 × 10^5^/well in white 96-well plates in complete DMEM medium without puromycin for 24 h. Then, puromycin-free medium was aspirated from the plates and replaced with 100 μL/well diluted Natural Product Collection library (1 μM or 0.1 μM) plates. Six wells on the sides were filled with 100 μL medium containing puromycin to be used as a reference control. The treated plates were transferred to a 37 °C CO_2_ incubator for 16 h, then transferred to a −80 °C freezer in order to lyse the cells. In order to measure luciferase activity, the LightSwitch™ Luciferase assay from SwitchGear genomics cat# LS010 was used. Briefly, 100 μL assay solution was added to each well and incubated for 30 min at room temperature in the dark, then each well was read for 2 s in a PerkinElmer Wallac Victor plate reader. The adjusted Foxp3 luciferase response units (RU) were calculated as RU = ((luminesce in test molecule × 100)/luminesce in medium) − 100.

### 2.4. Real-Time q-PCR

Promptly after collection, PBMCs were isolated from the buffy coat layer after density gradient centrifugation using Histopaque^®^-1077 (Sigma-Aldrich, Merck, St. Louis, MI, USA). Freshly isolated PBMCs were suspended in Complete RPMI-1640 (RPMI 1640 supplemented with 10% heat-inactivated fetal bovine serum, 2 mM L-glutamine, 100 μg/mL streptomycin, 100 U/mL penicillin, 50 mM HEPES, 0.15% NaHCO_3_ (*w*/*v*)), and treated with 10 ng/mL TGF-β, 5 μM sparteine sulfate or 1 μM harpagoside in the presence or absence of 5 μg/mL of the T cell mitogen ConA. On the fifth day of treatment, cells were harvested and RNA was isolated with the RNeasy kit (QIAGEN, Hilden, Germany). cDNA was synthesized using iScript Reverse Transcriptase Supermix (Bio-Rad, Hercules, CA, USA). Real-time quantitative PCR by SsoAdvanced Universal SYBR Green Supermix (Bio-Rad, Hercules, CA, USA) and specific primers to GAPDH, TGF-β, FoxP3 and IL-10 were used to amplify the cDNA for the cognate genes. GAPDH was used as an internal control, and the relative expression of TGF-β, FoxP3 and IL-10 was calculated using the 2^−ΔΔCt^ method. The primers were synthesized by Alpha DNA (Montreal, QC, Canada); primers sequences were as follows: GAPDH (F: 5′ AG-GGCTGCTTTTAACTCTGGT 3′, R: 5′ CCCCACTT-GATTTTGGAGGGA 3′), TGF-β1 (F: 5′ CACGTGGAGCTGTACCAGAA 3′, R: 5′ GAACCCGTTGATGTCCACTT 3′), IL-10 (F: 5′ TGCCTTCAGCAGAGTGAAGA 3′, R: 5′ GTCTTGGTTCTCAGCTTGGG 3′) and FoxP3 (F: 5′ CACTGCTGGCAAATGGTGTC 3′, R: 5′ GGATGGCGTTCTTCCAGGTG 3′). All genes were assessed in triplicate. At least three biological replicates using cells from three different donors were used.

### 2.5. Flow Cytometry

PBMCs prepared as described above were treated with 10 ng/mL TGF-β, 5 μM sparteine sulfate or 1 μM harpagoside in the presence or absence of 5 μg/mL of the T cell mitogen Con A. On the fifth day of treatment, cells were harvested and stained with FITC-conjugated anti-CD127 (351312), PE-conjugated anti-CD25 (356104) and PerCP/Cy5.5-conjugated anti-CD4 (344608) (Biolegend, San Diego, CA, USA). For the Foxp3 intracellular staining, Alexa Fluor^®^ 647 antihuman FOXP3 Antibody and True-Nuclear™ Transcription Factor Buffer Set were used according to the manufacturer’s protocol (Biolegend, San Diego, CA, USA). The cell population with double mean fluorescence intensity of CD25^+^ cells was defined as CD25^hi^ cells [19]. Compensation was performed by running single color controls with BD CompBeads (552843). After staining, cells were acquired in BD FACS-ARIA III (BD-Bioscience, Franklin Lakes, NJ, USA) using FACSDiva software and analysis was performed using FlowJo software (Tree Star, San Carlos, CA, USA). Singlet’s gating was used to avoid doublets; CD25, FOXP3 and CD127 FMO controls were used to set up the gates for T regulatory cells.

### 2.6. Cytokine Quantitation

The detection of the Treg cytokines IL-10 and TGF-β secreted from treated PBMCs was performed via ELISA. The supernatants from the cells in flow cytometry experiments described above were collected on the day of the harvest and kept at −80 °C. A sandwich of hIL-10 (E-EL-H0103) and hTGF-β (E-EL-H0110) ELISA kits was used according to the manufacturer’s instructions (Elabscience, Houston, TX, USA). Briefly, thawed undiluted PBMC culture supernatants from 3 biological replicates were added to coated kit wells in duplicates. The plates were read via a microtiter plate reader (BioTek Elx 808) at 450 nm, with Gen5 Imager software. The concentration of samples was determined using a 4-parameter logistic (4-PL) curve fitted via Graphpad Prisim 8.4.2.

### 2.7. Allogeneic Mixed Lymphocyte Reaction (MLR)

The proliferative response of T cells in PBMCs to allogeneic cells was measured by one-way MLR [29]. This was accomplished by mixing PBMCs from two different unrelated donors in the presence or absence of 5 μM SS or 5 μM Harp. The one-sided response was ensured by treating one of the donor’s PBMCs with 25 μg Mitomycin C for 1 h at 37 °C then washing 3 times in complete RPMI; such cells were considered stimulators. The one-way MLR was conducted as described by Kruisbeek et al. [30]. Briefly, in a 96-well plate, isolated PBMCs from two individuals were co-cultured; cells that served as stimulators were added at 0.6 × 10^6^/well while responder PBMCs were added at 0.4 × 10^6^/well. MTT colorimetric assay was used to measure the proliferative response of T cells. Absorbance was measured at 570 nm, and stimulation index (S.I.) was calculated follows: S.I. = Optical Density (OD) of responder cells in wells with stimulator cells added/OD of the same responders in wells containing responder cells only.

### 2.8. Statistical Analysis

All experiments were performed in triplets and represented as average ± standard error (S.E.). The Friedman test (a nonparametric one-way ANOVA on ranks) was used to assess significant differences between test and control (peripheral blood mononuclear cells (PBMC) in medium or with ConA). To do this, we used the statistical software GraphPad Prism 8.4.2. *P*-values < 0.05, <0.005 or <0.0005 were considered statistically significant and denoted with an asterisk, (*), (**) or (***), respectively.

### 2.9. Ethics Statement

This study was carried out according to the ethical guidelines of the Research Institute of Medical and Health Sciences (RIMHS) Research Ethics Committee (approval: REC-19-09-25-01-S). Human samples were obtained in accordance with the Medical Research Involving Human Subjects Act, and informed consent was obtained from all subjects.

## 3. Results

### 3.1. SS and Harp Induced the Expression of Luciferase in the FoxP3 Reporter Cell Line

**Figure 1:**
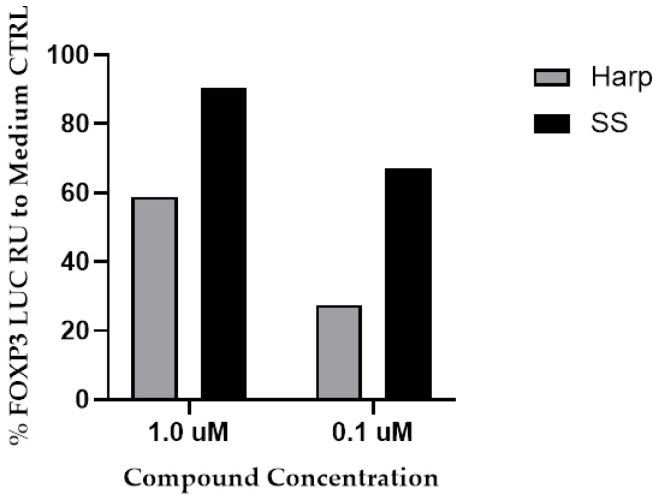
The induction of FoxP3 promotor by the hit molecules. Sparteine sulfate (SS) or harpagoside (Harp) (1 or 0.1 μM) was added to the HEK 293-FoxP3 reporter cell line for 16 h. Cells were then lysed, and luciferase activity was measured. Response units (RU) were adjusted in relation to response in wells with media + puromycin (CTRL). The % Foxp3 induction is calculated as [(luminescence for test molecule × 100)/luminescence for medium CTRL] − 100. The two library replicates of diluted plates were run on separate days, and there were no experimental replicates.

Due to the great potential of Treg cells in the downregulation of inflammatory diseases and the key role of FoxP3 expression in their induction, we screened a library of 800 small natural molecules for their ability to induce FoxP3 expression. To this end, we used the HEK 293 Human FoxP3 Prom/LUCPorter™ stable reporter cell line and screened the 800 small natural molecules for their ability to induce Luciferase activity. From the 800 molecules, only 50 were able to induce FoxP3 promoter above 50% as compared to medium control (CTRL) in a dose-dependent mater (data not shown). Among the top 50 hits, SS and Harp were chosen for this study due to their safety and the fact that both have relatively well-established medicinal uses. SS and Harp induced the expression of FoxP3 promoter at concentrations of 1 μM (90% and 59%, respectively) and 0.1 μM (67% and 28%, respectively), as shown in Figure 1.

### 3.2. Cytotoxicity of SS and Harp on PBMCs In Vitro

The assessment of SS and Harp cytotoxicity was conducted via XTT assay for cell viability. ConA-activated PBMCs were incubated in the presence or absence of serial doubling dilutions of SS and Harp for 48 h. There was no evident toxicity up to 200 μM for SS, and no toxicity was detected up to 100 μM of Harp (please see Supplementary Materials). Thus, both molecules are not toxic on in vitro grown PBMCs at the concentrations used throughout this study.

### 3.3. Harp and SS Upregulate Expression of mRNA for Treg Cell Markers In Vitro

**Figure 2:**
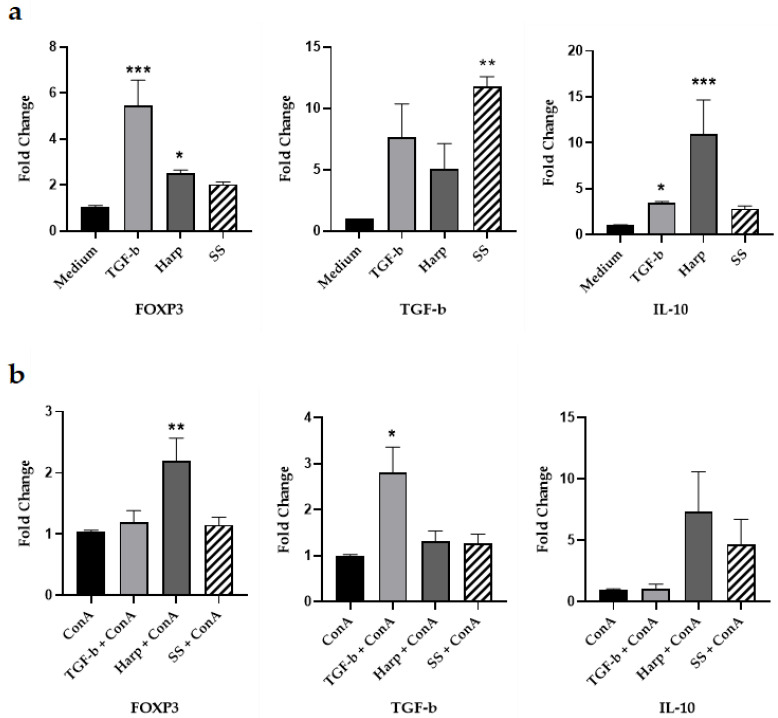
Changes in the FoxP3, IL-10 and TGF-β mRNA expression 5 days after addition of compounds. Peripheral blood mononuclear cells (PBMCs) were isolated and incubated without activation along with 10 ng/mL TGF-β, 1 μM Harp or 5 μM SS for 5 days. Fold difference in the expression of FoxP3, TGF-β and IL-10 mRNA: (**a**) Fold difference in the expression of mRNA in cells without ConA and (**b**) with ConA 5 μg/mL. Data shown are from one representative experiment out of three, showing fold change ± S.E. Statistical significance shown as expression compared with control PBMCs in medium alone for the non-activated PBMCs or with ConA alone for the activated PBMCs that are treated with Harp, SS or TGF-β cells (* *p* < 0.05, ** *p* < 0.005 and *** *p <* 0.0005). Data shown represent three biological replicates.

In order to test whether SS and Harp can actually induce expression of Foxp3 mRNA, PBMCs from healthy volunteers were grown in the presence or absence of SS or Harp, and TGF-β1 was used as a positive control. A group of wells was also activated with the T cell mitogen 5 μg/mL ConA. All cells were incubated for 5 days, then harvested and total RNA was extracted from each sample and subjected to reverse transcription and real time q-PCR. In addition to FoxP3, we assessed for the expression of key Treg-derived cytokines IL-10 and TGF-β. Data depicted in Figure 2 show the fold change in expressions of FoxP3, TGF-β and IL-10 mRNA, comparing the expression of these genes in the sample that contained medium alone (Figure 2a) with that in the sample activated with ConA (Figure 2b). In the unstimulated cells, TGF-β, Harp and SS all increased the expression of FOXP3 mRNA, yet significantly only in the presence of TGF-β or Harp with *p* = 0.0004 and 0.0110, respectively. The same is true for IL-10 mRNA, which was increased by TGF-β, Harp and SS, significantly only in TGF-β and Harp-treated cells with *p* = 0.0417 and 0.0002, respectively. The TGF-β mRNA was increased in the unstimulated cells in the presence of TGF-β, Harp and, SS but only significantly by SS with *p* = 0.0110. In the ConA-activated cells, Harp increased the expression of FOXP3 significantly with *p* = 0.0017; it also increased the expression of TGF-β and IL-10 but not significantly. SS resulted in a slight increase in the expression of the three genes’ mRNA but such an increase was not statistically significant. These data demonstrate that the two molecules were able to induce the expression of Foxp3 and the expression of key Treg cell cytokines TGF-β and IL-10 from naïve PBMCs, with a lesser level of increase in the ConA-activated PBMCs.

### 3.4. Harp and SS Increase Percentege of CD4^+^CD25^+^ Treg Cells in PBMCs In Vitro

**Figure 3:**
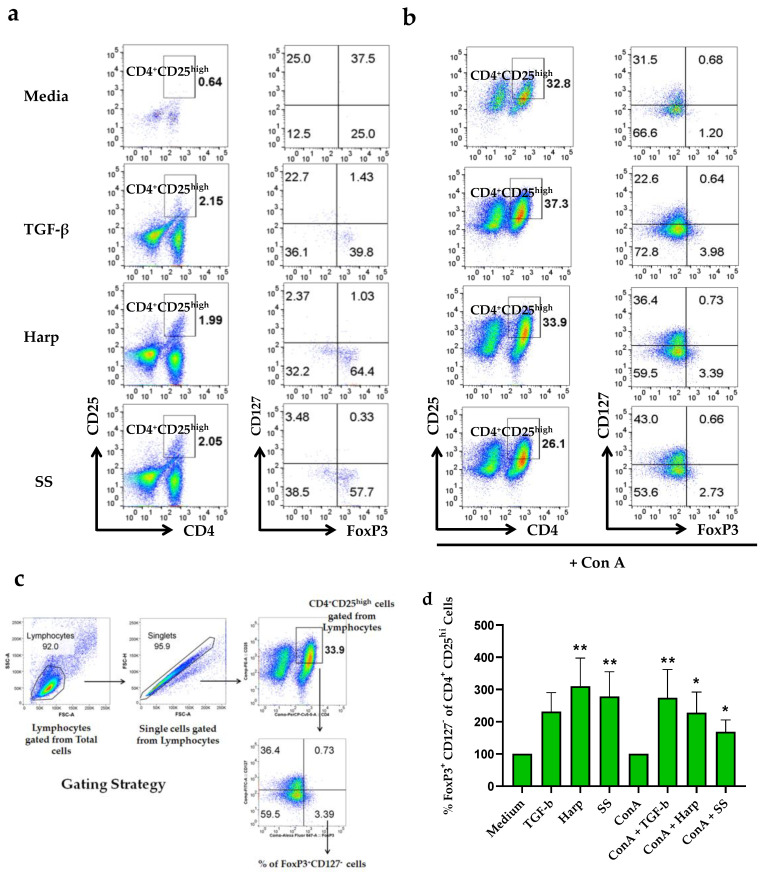
Flow cytometric analysis for the CD4^+^CD25^hi^FoxP3^+^CD127^−^ T cells. Human peripheral blood mononuclear cells were isolated and incubated in the presence or absence of 5 μg/mL ConA along with either 10 ng/mL TGF-β, 1 μM Harp or 5 μM SS for 5 days. (**a**) Effect of treatment with compounds in the absence of ConA; and (**b**) in the presence of ConA. (**c**) The gating strategy for CD4^+^CD25^hi^FoxP3^+^CD127^−^ T cells. (**d**) Percentages of FoxP3^+^CD127^−^CD4^+^CD25^hi^ T cells following various treatments, showing average of triplicate and calculated as % of total lymphocytes ± S.E. Statistically significant difference when % is compared with medium alone in non-activated cells or with ConA-activated PBMCs for the ConA-activated cells (* *p* < 0.05 and ** *p* < 0.005). The data shown in (**d**) represent two biological replicates.

To determine whether Harp and SS can affect the phenotypic profile of T cells, PBMCs isolated from donors’ blood were grown in the presence or absence of either one of the compounds, in similar combinations as in the mRNA experiments described above, then harvested and stained with fluorescent antibodies against CD4, CD25, CD127 and FoxP3. Tregs were identified based on their higher expressions of the surface markers CD25(CD4^+^CD25^hi^) and CD127^−^FoxP3^+^ [29]. To this effect, CD4^+^CD25^hi^ double positive cells were gated on and checked for the number of FoxP3^+^CD127^−^ cells as shown in Figure 3a–d. Data showed that both Harp and SS when paired with ConA T cell activator significantly increased the percentage of cells with Treg cell profile as compared with cells with ConA alone, with *p* = 0.0417 and 0.0417, respectively (Figure 3b,d). Interestingly, Harp and SS both induced significantly increased percentages of T cells with Treg profile even in the naïve and unstimulated cells (with *p* = 0.001 and 0.0076, respectively) (Figure 3a,d). In addition, the incubation with recombinant TGF-b increased the % of T cells with Treg profile in the presence or absence of ConA; this increase was significant only in the ConA-activated cells, *p* = 0.0052.

### 3.5. SS and Harp Induced the Production of the Anti-Inflammatory Cytokine IL-10

**Figure 4:**
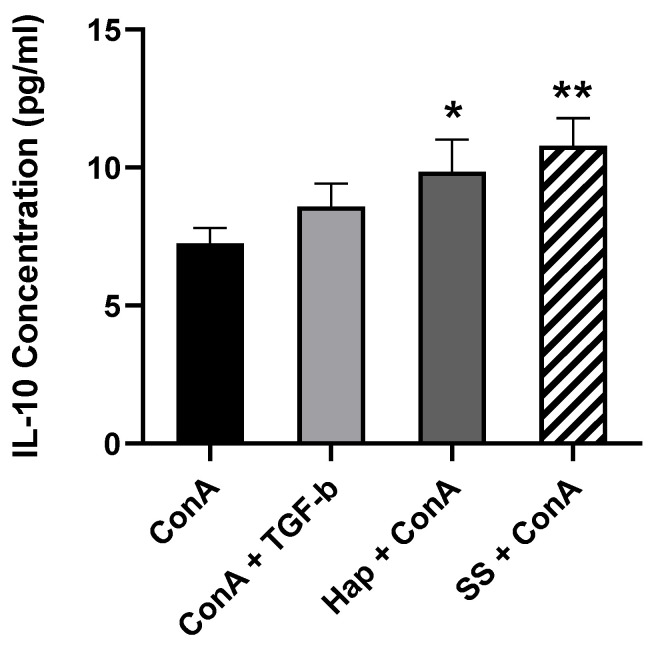
The secretion of IL-10 by induced SS and Harp-treated PBMCs in vitro. After 5 days of incubation in the presence of 5 μg/mL ConA along with either 10 ng/mL TGF-β, 1 μM Harp or 5 μM SS, undiluted supernatant samples were analyzed via sandwich ELISA to assess IL-10 content. Values plotted are the average of duplicate wells ± S.E. Statistically significant differences compared to ConA-activated PBMCs are indicated with * *p* < 0.05 and ** *p* < 0.005. Data shown represent 3 biological replicates.

IL-10 and TGF-β are the signature cytokines for functional Tregs, and for that reason, their presence in the supernatants of the cultured cells was assessed. This was done by incubating PBMCs in the presence or absence of 5 μg/mL ConA and either 10 ng/mL TGF-β, 1 μM Harp or 5 μM SS for 5 days. At the end of incubation, cells were harvested and the supernatants were passed through a 0.2 μm filter in preparation for ELISA. The data shown in Figure 4 demonstrate that the treatment of ConA-activated PBMCs with Harp and SS resulted in a statistically significant increase in the amount of IL-10 produced (*p* = 0.0235 and *p* = 0.0017, respectively). In the wells without ConA, there was no detectable IL-10 (data not shown). The same supernatants were tested for the content of TGF-β via sandwich ELISA; none was detected in the supernatants despite the increase in TGF-β mRNA (please see discussion). These results suggested that induction of CD4^+^CD25^+^ Treg cells by SS or Harp results in secretion of higher levels of IL-10 in activated PBMCs.

### 3.6. Harp and SS Are Able to Suppress T Cell-Mediated Allogeneic MLR

**Figure 5:**
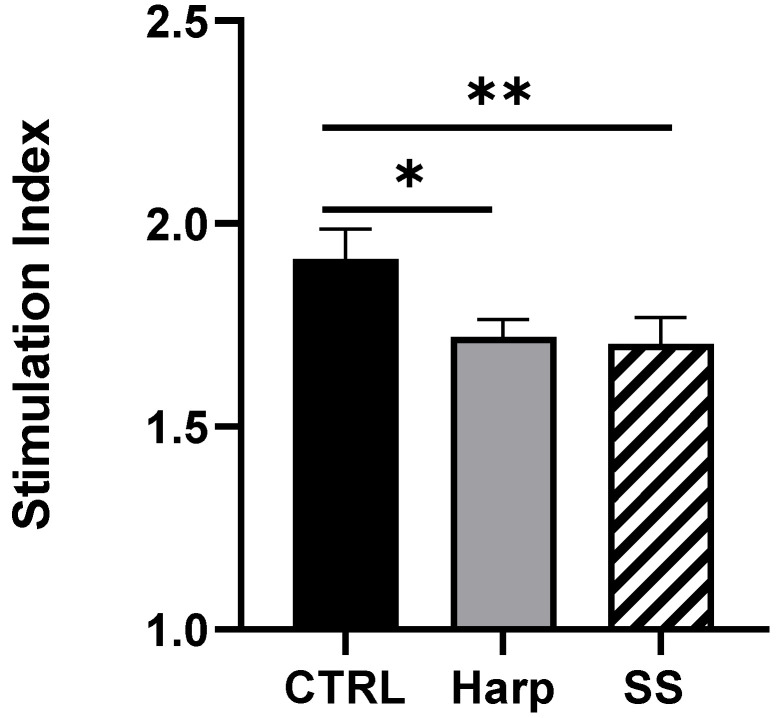
Allogeneic mixed lymphocyte reaction (MLR) in the presence or absence of 5 μM of each of Harp and SS. Average simulation index of triplicates is plotted ±S.E. Statistically significant differences compared with untreated CTRL MLR are indicated with asterisks (* *p* = 0.0578 and ** *p* = 0.0044, respectively). The data shown represent two biological replicates.

In order to obtain a direct indication of the immunosuppressive effect of the two compounds, one-way MLR was conducted. The co-culturing of PBMCs from two different volunteers in the presence of SS or Harp resulted in a significant reduction in the proliferative response in allogeneic activated T cells (*p* = 0.0578 and 0.0044, respectively). The reaction was repeated twice with two responders’ peripheral blood mononuclear cells (PBMCs); the data shown in Figure 5 are from one representative experiment. The reduction implies that the CD4^+^CD25^+^ Treg cells induced by SS or Harp are capable of suppressing antigen-specific responses.

## 4. Discussion

Anti-inflammatory agents are essential in the control of autoimmune and chronic inflammatory diseases. Treg cells are key players in the regulation of inflammatory responses, and the search for new agents that can induce Tregs is gaining increasing interest. In the study herein, we present two small natural molecules, sparteine sulfate and harpagoside, as potential anti-inflammatory agents due to their ability to induce Tregs in vitro. The two molecules were able to induce expression of the FoxP3 transcriptional factor in vitro as shown through mRNA expression and flow cytometry. This induction was measured in total PBMCs in the presence or absence of the T cell mitogen ConA; such an approach may allow for cell–cell contacts and is closer to in situ conditions than if we had used isolated activated T cells. The signature Treg cell cytokines IL-10 and TGF-β were also upregulated to a varying extent as evidenced by q-PCR and ELISA. The fact that TGF-β was not detected by ELISA can be attributed to the fact that TGF-β may be produced at a very low concentration and that it is often thought to be sequestered by binding to extracellular matrix components [31,32]. TGF-β has been shown to upregulate expression of FoxP3 and induce differentiation of CD4^+^CD25^−^ precursors into suppressive Tregs cells [31]; therefore, samples treated with TGF-β are considered an appropriate reference for comparison. For IL-10 expression, the amount of IL-10 produced by non-activated cells was not detected via ELISA, despite the fact that the mRNA showed upregulation in IL-10. This discrepancy can be explained by the fact that at the end of incubation in the wells without ConA, some cells may have died due to a lack of antigen receptor signal. Such an observation was described by Rathmell et al., 2000, where it was shown that activation through cell-specific receptors promotes cell survival by regulating nutrient uptake and utilization [33]. In addition, unlike cells that were activated with ConA, there was no growth or proliferation. Consequently, from cells without ConA, we obtained less total RNA than that which was extracted from their ConA-activated counterparts. Nevertheless, when running the q-PCR, we unified the amount of starting RNA from all samples for the purposes of comparison. Hence, the discrepancy is due to the fact that q-PCR data represent the expression per 0.5 μg of RNA, while the ELISA data represent IL-10 secreted from all the cells in each well. Haller et al. reported a similar observation in unstimulated PBMCs where expression of IL-10 was detected via q-PCR and none through ELISA [34].

Tregs are generally classified into two categories: either natural (thymus-derived) (nTregs) or induced (peripherally derived) (iTregs). Natural Tregs represent between 2% and 8% of CD4^+^ T cells in healthy donor peripheral blood, whereas induced Tregs can be generated by the expansion of CD4^+^ T cells in the presence of TGF-β [11,35]. In this study, the percent of CD4^+^CD25^+^FoxP3^+^CD127^−^ Tregs was shown to be enhanced by the TGF-β and the two compounds Harp and SS. This has been shown in both the presence and absence of antigenic/mitogenic stimulation. The numbers presented in flow cytometry represent a % of 1 × 10^6^ cells; however, it does not reflect the total number of CD4^+^CD25^+^FoxP3^+^CD127^−^ Treg cells per well. The wells with ConA had 3–4 times the number of cells in the non-activated wells; thus, there were far more cells with Treg profile in the ConA-activated wells. The origin of these Tregs (i.e., whether they are nTregs or iTregs) was not within the scope of this investigation. Future studies may analyze the origin and mechanism by which such Tregs are induced by Harp and SS.

Our results indicate that both sparteine sulfate and harpagoside can induce an increase in T cells with Treg profile. Most important is the ability of both molecules to reduce the allogeneic specific responses in MLR. The induced reduction of response in the MRL assay could be attributed to the fact the both harpagoside and sparteine sulfate were able to increase Treg cells in vitro even from naïve cells. Furthermore, it was interesting to note that both were able to increase expression of IL-10 and TGF-β mRNA in both activated and non-activated cells. IL-10 and TGF-β have been shown to suppress allogeneic MLR in recent studies [36,37,38].

While harpagoside and devil’s claw extract have been used in folk and contemporary medicine as anti-inflammatory agents, the current study is the first to demonstrate harpagoside’s ability to induce FoxP3 and the subsequent differentiation of CD4^+^CD25^+^ Treg cells in human PBMCs. Huang et al. showed that Harp inhibited the Lipopolysaccharide (LPS)-induced activation of the transcriptional factor NF-κB promoter activity in a gene reporter assay in the macrophage cell line RAW 264.7 [39]. The NF-κB transcriptional factor is involved in the expression of many human inflammatory cytokines including but not limited to IL-1, 2, 8, 6 and 15, TNF-α and several chemokines. Such inhibitory effects may synergize with its ability to induce FoxP3 activation and Treg cell activation as shown in this study, thus making harpagoside a very convincing contender as an anti-inflammatory drug.

Sparteine sulfate, on the other hand, is already a drug that has been indicated as a class 1a antiarrhythmic agent as well as an oxytocic drug that stimulates contraction of the myometrium. There are no studies to our knowledge that link it to any immunosuppressive activity. Thus, this study demonstrates for the first time that sparteine sulfate may have great potential as an anti-inflammatory agent due to its evidenced ability to induce Tregs from naïve or activated human PBMCs.

Together, our data indicate that sparteine sulfate and harpagoside may serve as agents for upregulating FoxP3 expression and driving cells towards a regulatory T cell phenotype, as well as suppressing the effect of effector T cells. Although these two compounds may have great potential as therapeutic agents for autoimmune, transplant rejection and chronic inflammatory conditions, further investigations are required in vivo to determine the efficiency of the compounds in inflammatory disease models.

## 5. Conclusions

In conclusion, we have demonstrated that harpagoside and sparteine sulfate, which are naturally occurring molecules derived from plants, are able to increase the expression of the transcription factor FoxP3. Furthermore, we have shown that naive PBMCs are induced to differentiate into a T regulatory phenotype that produces anti-inflammatory cytokines. These findings may have significant impacts on therapeutic treatments for autoimmune diseases, chronic inflammatory conditions and suppression of graft rejection. Additional studies are necessary in vivo to test the efficacy of these molecules in the induction of Tregs and suppression of inflammatory diseases.

## Supplementary Materials

The following are available online at https://www.mdpi.com/2079-7737/9/8/211/s1, Figure S1: XTT—Cytotoxicity assay showing cell viability of PBMC in vitro following treatment with doubling dilutions of the molecules being investigated

## Abbreviations

SS	Sparteine Sulfate
Harp	Harpagoside
PBMCs	Peripheral Blood Mononuclear Cells
ConA	Concanavalin A
FoxP3	Fork Head Box P3
TGF-β	Transformation Growth Factor-β
IL-10	Interleukin-10
MLR	Mixed Lymphocyte Reaction
