# Supplementary material for: Induction of CD4+CD25+ Regulatory T Cells from In Vitro Grown Human Mononuclear Cells by Sparteine Sulfate and Harpagoside
Source: Biology (Basel). 2020 Aug 6;9(8):211. doi: 10.3390/biology9080211 (PMC7464273; doi:10.3390/biology9080211)
Supplement: Supplementary file 1 [file biology-09-00211-s001.pdf]

A

B

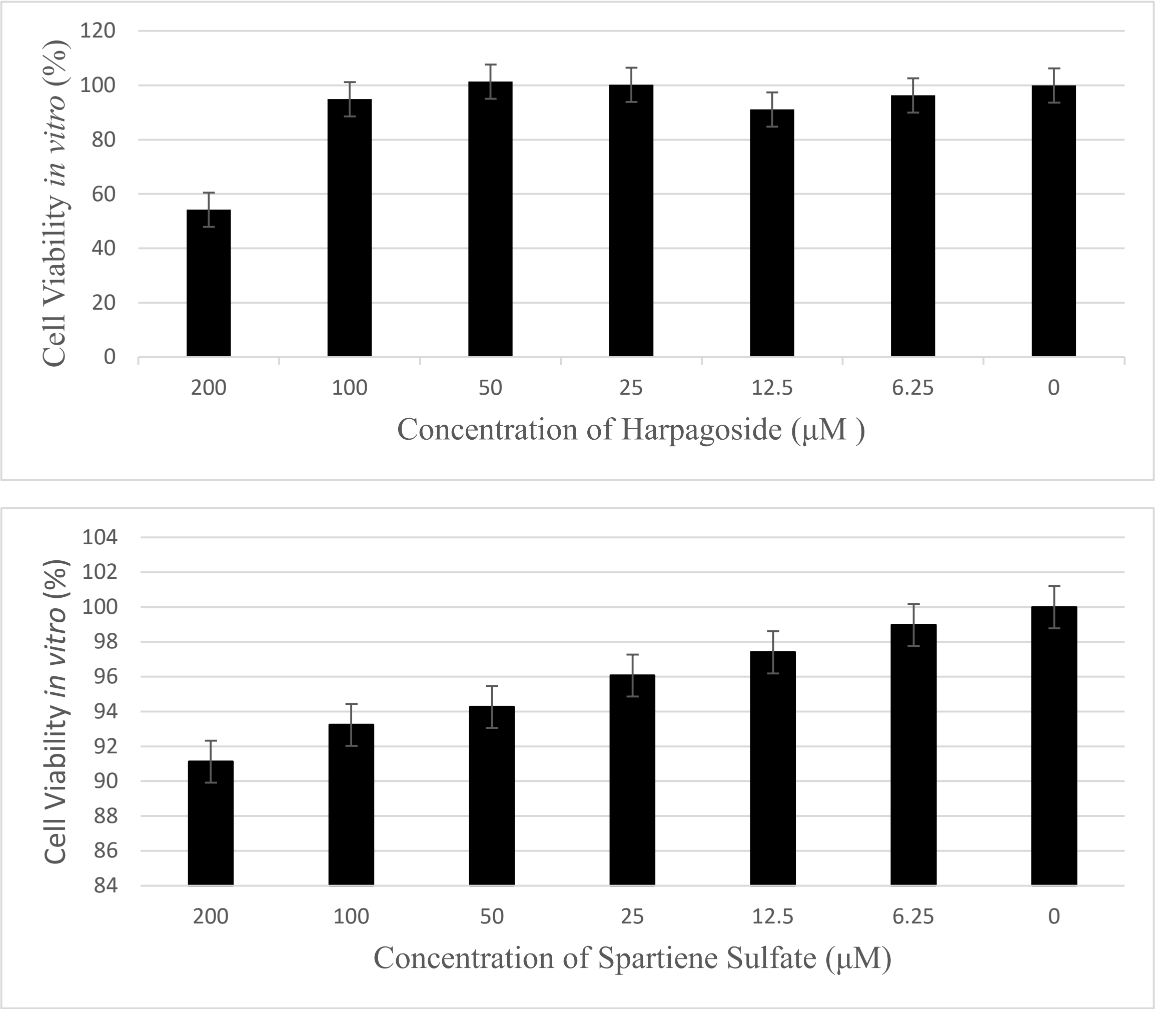

Figure S1. **XTT -Cytotoxicity assay showing cell viability of PBMC *in vitro* following treatment with doubling dilutions of the molecules being investigated**. (A)Percentage of PBMC viability after treatment for 48 hours with different concentrations of Spartiene Sulfate. (B) Percentage of PBMC viability after treatment for 48 hours with different concentration of Harpagoside. Average % of cell viability of triplicates is plotted ± S.E.
